# Assessment of Clinical Outcomes in Patients Presenting with Transverse Myelitis: A Tertiary Care Experience from a Developing Country

**DOI:** 10.7759/cureus.4342

**Published:** 2019-03-29

**Authors:** Aijaz Ali, Syeda Beenish Bareeqa, Amir Riaz, Syed Ijlal Ahmed, Muhammad Hassan Shaikh, Muhammad Ishaq Ghauri

**Affiliations:** 1 Neurology, Jinnah Medical College Hospital, Karachi, PAK; 2 Oncology, Jinnah Medical and Dental College, Karachi, PAK; 3 Rheumatology, Liaquat National Hospital and Medical College, Karachi, PAK; 4 Internal Medicine, Liaquat National Hospital and Medical College, Karachi, PAK; 5 Neurology, Aga Khan University and Hospital, Karachi, PAK; 6 Internal Medicine, Jinnah Medical College Hospital, Karachi, PAK

**Keywords:** transverse myelitis, neuromyelitis optica, multiple sclerosis

## Abstract

Background: Transverse myelitis (TM) is an inflammatory disorder of spinal cord, characterized by acute or sub-acute dysfunction of spinal cord affecting the motor, sensory, and autonomic systems. It may be idiopathic or related to other diseases. Although some patients recover from TM with minor or no residual problems, others suffer permanent impairments that affect their ability to perform ordinary tasks of daily living. Our objective was to determine the frequency of different clinical outcomes in patients presenting with TM.

Methods: It was a prospective cohort clinical study conducted from May 2018 till October 2018. Study was conducted in the Department of Neurology at Jinnah Medical College Hospital (JMCH), Karachi. In total 131 patients of TM were enrolled and treated as per standard protocol, and re-evaluated after eight weeks for assessment of clinical outcomes.

Results: The average age of patients was 51.15 ± 6.56 years. Out of 131 cases, 36.6% of patients had full recovery and 63.4% had poor recovery while recurrence occurred in 66.7% cases. Urinary frequency was observed in 12.2% cases and incontinence in 6.9% cases.

Conclusion: Acute TM has become transformed with recent developments, especially the advent of the MRI and the discovery of biomarkers.

## Introduction

Transverse myelitis (TM) is an inflammatory disorder of spinal cord, characterized by acute or sub-acute dysfunction of spinal cord affecting the motor, sensory, and autonomic systems. It may be idiopathic or related to other diseases. Lesions may occur at any level of the spinal cord but more commonly at the thoracic level [1].

No familial predisposition is apparent. There is a bimodal distribution with peak incidence among ages of 10-19 years and 30-39 years [2]. Symptoms of TM include sudden onset of lower back pain, muscle weakness, or abnormal sensations in the toes and feet which can rapidly progress to more severe symptoms, including paralysis, urinary incontinence, increased urinary urge, urinary incontinence, difficulty in urinating, and loss of bowel control [3]. The spinal cord MRI will almost always confirm the presence of a lesion within the spinal cord, whereas the brain MRI may provide clues to other underlying causes, especially multiple sclerosis. If an MRI is not possible, physicians may consider other diagnostic tests like CT scan of spine with or without myelography [4-5].

As with many disorders of spinal cord, no effective cure currently exists for people with TM. Treatments are designed to reduce spinal cord inflammation and manage and alleviate symptoms. Corticosteroid medications that might be prescribed may include intravenous methylprednisone or dexamethasone. In severe cases that do not appear to respond to corticosteroid treatment, other therapies such as plasma exchange or drug therapies may be used to try to salvage neurological function [6].

Recovery from TM usually begins within two to 12 weeks and may continue for up to two years. However, if there is no improvement within the first three to six months, complete recovery is unlikely. Although some patients recover from TM with minor or no residual problems, others suffer permanent impairments that affect ability to perform ordinary tasks of daily living [7]. Commonly experienced permanent neurological deficits resulting from TM include severe weakness, spasticity or paralysis, incontinence and chronic pain. A very recent study documented that about 13% patients of TM converted to multiple sclerosis; other 9.4% of patients with TM were completely paralyzed. The study found that 46.5% had urinary sphincter dysfunction [8].

Transverse myelitis is not a very uncommon disease in Pakistan. We frequently diagnosed such cases at our institute which provides healthcare to a very large population from urban as well as rural areas. Despite the frequent cases TM has not yet been studied in our population. There is utmost need to assess the various outcomes which happen in our TM patients so we can provide them with accurate and better care at an early stage in order to prevent neurological complication and undue morbidity. The current study is proposed with this background rationale. The results of this study will also increase our understanding regarding different sequel of TM in our population which has not yet been documented.

Our primary objective was to determine the frequency of different clinical outcomes in patients presenting with TM in a tertiary care center. 

## Materials and methods

We conducted a prospective cohort study from May 2018 till October 2018. It was conducted on 131 patients who presented as outpatient cases at the Department of Neurology, Jinnah Medical College Hospital (JMCH), Karachi.

Calculation of sample size was conducted by taking the expected least frequency of complete paralysis at 9.4% [8] in TM patients, level of significance as 95%, margin of error at 5% (α = 0.07) and using the WHO sample size calculator; the sample size calculated was 131 TM patients at least. Nonprobability, consecutive sampling technique was used for allocation of cases.

Inclusion criteria

· Patients of either gender; and age >30 years to <60 years with TM diagnosis

· TM was labeled when all following are present;

A. Presence of following clinical sign/symptoms:

a. Acutely developing paraparesis was said when a patient presents with the history of developing all following symptoms over 30 minutes to seven days:

 i. Flaccid paraplegia: power = 0/5 in affected limbs

 ii. Belt-like or radicular pain

 iii. Disturbed function of bladder/rectum

 iv. Isolated loss of pain and temperature sensation clinically detectable at clearly defined sensory spinal segmental level

 v. Intact while vibration sense and proprioception clinically detectable at clearly defined sensory spinal segmental level

b. No clinical evidence of spinal cord compression.

B. Confirmation of TM through MRI when all of the following are found positive;

a. Lesion of >50% of cross sectional area on axial scan of spinal cord

b. Sagittal T2-weighted image showing disc bulge and hyperintense lesion within spinal cord

c. Sagittal T1-weighted image after IV gadolinium administration showing ring enhancing lesion.

Exclusion criteria

· Any acute or past history of back trauma

· Progressive spastic paraparesis (patchy sensory deficit or hemicord syndrome)

· Gullain Barre syndrome, stroke, Alzheimer’s disease, encephalitis, meningitis or brain abscess, metastatic cancer to spinal cord or brain, multiple myeloma, vascular malformation (dural AV fistula, AVM, cavernous angioma), hepatic or uremic encephalopathy, syphilis

· Taking corticosteroid therapy within six months or neuromuscular blocking agents, history of depressive illness before the onset of TM.

After taking permission from the Ethical Review Committee of JMCH, the data collection was started. Patients presenting at the neurology ward with symptoms suggestive of TM which later confirmed through MRI were enrolled in the study after taking the written consent. The data on demographic variables including name, age, gender, residence, and duration of symptoms were noted on designed Performa. A thorough history and clinical examination was done by the researcher himself supervised by the consultant neurologist. Patients were treated as per standard protocol and were re-evaluated on follow-up at the eighth week. Data regarding the clinical outcome, i.e., full recovery (TM patients experience monophasic disease and have no evidence of multi systemic or multiphasic disease, restoration of neurological functions within eight weeks) [9], recurrence (persistent TM symptoms after the eighth week), neuromyelitis optica, multiple sclerosis (limb weakness: power < 4/5 in affected limb, sensory loss: loss of pain, temperature, vibration and proprioception at sensory spinal segmental level, diplopia; patient complaining of seeing two of same objects for >24 h and MRI shows oval-shaped lesions in <2 spinal cord segment with hyperintense shadow on T2 with homogenous or ring enhancement) [8], complete paralysis (patient having power 0/5 in all four limbs) [8], or absence of urinary sphincter control (urinary frequency: urinates within <2 h of urination or incontinence: complaints of involuntary leaking/passage or urine while doing normal activities) [10] was noted in TM.

The data were analyzed using the SPSS version 21.0. The age and symptoms of disease were expressed in mean ± standard deviation (SD) values. Gender, residence, and various clinical outcomes (full recovery, recurrence, neuromyelitis optica, multiple sclerosis, complete paralysis or absence of urinary sphincter control-urinary frequency or incontinence which are the outcome variables) were presented by their frequencies along with percentages. Age, gender, residence, and duration of symptoms were stratified into categories followed by application of chi-square test to evaluate their effect on the outcome variable. p ≤ 0.05 was considered as significant. 

## Results

The average age and duration of symptoms were 51.15 ± 6.56 years and 12.23 ± 6.23 days. There were 62 (47.33%) males and 69 (52.67%) females. Most of the cases were rural. The descriptive statistics about baseline data is given in Table 1.

**Table 1:** Baseline characteristics of transverse myelitis (TM) patients.

Characteristics	Distribution of variables
Age (years)	51.15 ± 6.56
Age ≤40 years	10 (7.6%)
Age 41-50 years	46 (35.1%)
Age 51-60 years	75 (57.3%)
Male/Female	62 (47%) / 69 (53%)
Duration of symptoms (days)	12.23 ± 6.23
Residence (Rural/Urban)	90 (69%) / 41 (31%)

Frequency of different clinical outcomes in patients presenting with TM at the eighth week follow-up was assessed. Out of 131 cases 36.6% of patients had full recovery with the ability to walk, and 63.4% had poor recovery with significant neurological deficits while recurrence occurred in 66.7% (32/48) cases. Some 75(90.4%) cases likely required further work up and close monitoring over time for multiple sclerosis, neuromyelitis optica and 9.6% patients with TM were completely paralyzed. Out of 131 cases, urinary frequency was observed in 12.2% (16/131) cases and incontinence was observed in 6.9% (9/131) cases. Summary of all the outcomes is presented in Table 2.

**Table 2:** Clinical outcomes of transverse myelitis (TM) patients after treatment.

Clinical outcome	Frequency	Percentage (%)
Full recovery	48	36.6
Recurrence	32	66.7
Neuromyelitis optica	41	49.4
Multiple sclerosis	34	41.0
Completely paralysis	08	9.6
Absence of urinary sphincter control		
Urinary frequency	16	12.2
Incontinence	9	6.9

No significant difference (p => 0.05) in clinical outcomes was found when compared with respect to baseline variables (age, gender, residence, and duration of symptoms).

## Discussion

Acute TM is a pathogenetically heterogeneous inflammatory disorder of the spinal cord. An incidence of 1.4 per 100,000 has been reported in Western literature [11]. TM has been reported as the major cause of noncompressive myelopathy [12-13]. A variety of disorders can cause acute TM and includes infections, para and post infections, and vascular, neoplastic, paraneoplastic, collagen vascular, and iatrogenic irregularities [14].

In our study, out of 131 patients with TM, the average age was 51.15 ± 6.56 years. There were 62 (47.33%) males and 69 (52.67%) females. This female predominance was also observed by Calvo et al., who found that, among the 85 acute TM patients included in the study, 53% were women and mean age at onset was 43 ± 16.2 years [8]. Two recent studies did find a higher incidence in females but an important caveat to one of these studies is that the proportion of cases that later were diagnosed as multiple sclerosis was not specified [15-16].

According to studies regarding idiopathic TM, ultimately 33% of patients have full recovery with the ability to walk, one-third have a fair recovery with some deficits, and 34% have poor recovery with significant neurological deficits [9, 17]. Relapses or recurrences can happen. One study found that about 39% of patients remained monophasic, while recurrences occurred in 61% of cases [15].

In these cases patients will likely require further work up and close monitoring over time for multiple sclerosis and neuromyelitis optica. A study reported that 23.5% patients converted to neuromyelitis optica [15]. Knebusch et al. estimated that a good outcome with normal gait occurred in 44%, a fair outcome with mild spasticity occurred in 33%, and a poor outcome with the inability to walk or severe gait disturbance and absence of sphincter control in 23% [10]. It has been highlighted that early recognition of neuromyelitis optica and other outcomes of TM is extremely important for prevention of serious neurological deficits [18].

Recurrences are unusual in acute TM compared to multiple sclerosis [19-20]. Some patients have lasting motor, sensory, or urological complaints. Persistent genitourinary symptoms include detrusor hyperreflexia, detrusor-external sphincter dyssynergia, and erectile dysfunction [21]. In our study out of 131 cases, urinary frequency was observed in 12.2% cases and incontinence was observed in 6.9% cases.

The incidence of multiple sclerosis in TM ranges from 0% over 6.5 years [22], 1% over 15 years [11], 6% over five years [23], 3% after five years [24], 3% over six years [25], or 8%-14% after an average of five years [26], 13% over 4.5 years-four fifths had brain lesions at presentation [5], 29% over five years [27], 40% of 20 patients over 12-30 months [28] or 45% over six years [29]. The second attack, i.e., multiple sclerosis, is the most likely to occur one year later and unlikely if two years have passed [27]. In light of our findings and literature available, out of 131 cases in our study 90.4% likely required further work up and close monitoring over time for multiple sclerosis and neuromyelitis optica.

The sequelae of TM are variable, even after severe paralysis. One third of patients recover completely, one third does not improve, and one third has some degree of residual symptoms [24, 26]. Patients with multiple sclerosis-like acute partial myelitis have a better short-term prognosis and are more likely to recover than patients with acute complete TM. International studies define a patient population with a low conversion rate to multiple sclerosis [30]. Our study confirms that 63.4% have poor recovery with significant neurological deficits while recurrence occurred in 66.7% (32/48) cases.

Early studies reported that up to one third of patients with a first episode of acute TM remained unable to walk. Most studies disclosed similar rates of poor outcome ranging from 11% to 35.8% [15]. In our series, only 9.4% of patients were unable to walk unassisted at completion of follow-up. In our study out of 131 cases, 63.4% cases have poor recovery with significant neurological deficits and 9.6% patients with TM were completely paralyzed.

## Conclusions

We conclude that acute TM has transformed with recent developments, especially the advent of the MRI and the discovery of biomarkers. It is the clinician’s responsibility to label acute TM according to internationally established criteria, starting with a clinical description of partial or complete TM. Imaging studies should be done in the acute phase of the illness so as to accurately measure the length of spinal cord lesions and should include the brain as well. A long-term follow-up study is of utmost need to assess the various outcomes which happen in our TM patients so that we can provide them with accurate and better care at an early stage in order to prevent neurological complication and undue morbidity.
